# Genome-wide Methylation Dynamics and Context-dependent Gene Expression Variability in Differentiating Preadipocytes

**DOI:** 10.1210/jendso/bvae121

**Published:** 2024-06-27

**Authors:** Binduma Yadav, Dalwinder Singh, Shrikant Mantri, Vikas Rishi

**Affiliations:** Nutritional Biotechnology, National Agri-Food Biotechnology Institute, Mohali, Punjab 140306, India; Regional Center for Biotechnology, Faridabad, Haryana 160014, India; Nutritional Biotechnology, National Agri-Food Biotechnology Institute, Mohali, Punjab 140306, India; Department of Anatomy and Cell Biology, Western University, London, Ontario N6A 5C1, Canada; Nutritional Biotechnology, National Agri-Food Biotechnology Institute, Mohali, Punjab 140306, India; Nutritional Biotechnology, National Agri-Food Biotechnology Institute, Mohali, Punjab 140306, India

**Keywords:** DNA methylation, context-dependent gene expression, differentially methylated regions, hotspots, obesity, Whole Genome Bisulphite Sequencing, 3T3-L1, obesity, and adipogenesis

## Abstract

Obesity, characterized by the accumulation of excess fat, is a complex condition resulting from the combination of genetic and epigenetic factors. Recent studies have found correspondence between DNA methylation and cell differentiation, suggesting a role of the former in cell fate determination. There is a lack of comprehensive understanding concerning the underpinnings of preadipocyte differentiation, specifically when cells are undergoing terminal differentiation (TD). To gain insight into dynamic genome-wide methylation, 3T3 L1 preadipocyte cells were differentiated by a hormone cocktail. The genomic DNA was isolated from undifferentiated cells and 4 hours, 2 days postdifferentiated cells, and 15 days TD cells. We employed whole-genome bisulfite sequencing (WGBS) to ascertain global genomic DNA methylation alterations at single base resolution as preadipocyte cells differentiate. The genome-wide distribution of DNA methylation showed similar overall patterns in pre-, post-, and terminally differentiated adipocytes, according to WGBS analysis. DNA methylation decreases at 4 hours after differentiation initiation, followed by methylation gain as cells approach TD. Studies revealed novel differentially methylated regions (DMRs) associated with adipogenesis. DMR analysis suggested that though DNA methylation is global, noticeable changes are observed at specific sites known as “hotspots.” Hotspots are genomic regions rich in transcription factor (TF) binding sites and exhibit methylation-dependent TF binding. Subsequent analysis indicated hotspots as part of DMRs. The gene expression profile of key adipogenic genes in differentiating adipocytes is context-dependent, as we found a direct and inverse relationship between promoter DNA methylation and gene expression.

DNA methylation is a fundamental epigenetic mechanism that plays a vital role in cell differentiation, a process by which an undifferentiated cell becomes a more specialized cell type with specific functions and characteristics [1-4]. DNA methylation is a chemical modification that involves adding a methyl group (CH_3_) to the cytosine base of CpG dinucleotide, where guanine succeeds cytosine [4-7]. This modification typically occurs at the 5′ carbon of the cytosine ring, and it is performed by DNA methyltransferases (DNMTs) [8]. CpG sites are found throughout the genome, and their methylation status can be heritable, allowing the epigenetic information to be transmitted from 1 cell generation to the following [9]. The consequences of its dysregulation further underscore the importance of DNA methylation in cell differentiation. Abnormal DNA methylation patterns have been implicated in various developmental disorders and diseases, including cancer [7, 10, 11]. Hypomethylation, the loss of DNA methylation, can lead to the reactivation of silenced genes, potentially causing cells to revert to an undifferentiated state or exhibit uncontrolled growth. Alternatively, hypermethylation, the excessive methylation of CpG sites, can result in the inappropriate silencing of critical genes, disrupting normal cellular differentiation processes [10-14].

A molecular mechanism is proposed on how DNA methylation in gene promoter regions can act as an active or repressive mark, allowing or inhibiting the binding of transcription factors and other regulatory proteins necessary for gene activation [15-17]. Conversely, DNA methylation in gene body regions is generally associated with gene activation. This process, known as gene body methylation, is less understood but appears to play a role in enhancing transcriptional elongation and stabilizing gene expression levels [18, 19].

DNA methylation plays a remarkable role in adipogenesis as in many physiological processes, the process in which preadipocytes (Pre-AD; undifferentiated cells) develop into mature adipocytes (fat cells). During adipogenesis, multipotent mesenchymal stem cells differentiate into mature adipocytes through tightly regulated molecular events. The orchestration of this process involves epigenetic alterations such as DNA methylation, modifications in histones, and the regulation by noncoding RNA [20]. Previous studies have shown that dynamic alterations in DNA methylation patterns occur during adipogenesis, affecting the expression of genes associated with adipocyte development, lipid metabolism, and adipose tissue function. Gene expression is initiated by the transcription factor's binding to the cis-elements in the promoter. DNA methylation of CpG dinucleotides profoundly affects the transcription factor binding to cognate cis-elements in promoters. In most cases, cytosine methylation in CpG dinucleotide is a repressor marker since transcription factors cannot bind to methylated sequences, leading to downregulation of gene expression. Previously, it was reported that at the initial stages of differentiation, there are prominent changes in overall methylation at promoters of adipogenic transcription factors such as PPARγ and C/EBPα. At the onset of mature adipocyte formation, the promoter methylation decreases [21, 22]. Similar observations were made for adipogenic genes like AdipoQ, adiponectin, and leptin, where promoter hypermethylation downregulates expressions [21, 23, 24]. However, in a few cases, the reverse is true, where methylation of CpG leads to gene expression. In earlier studies, it is shown that CREB fails to bind to CRE and CRE-like methylated sequences present in the promoters of adipogenic genes. In contrast, it is well bound by C/EBPα and β, both pivotal in adipogenesis [25]. In addition to promoter region, methylation of untranslated (UTR) regions also dictates gene expression. For example, hypermethylation of the 3′ UTR region of the adipokine gene results in its secretion disruption, whereas, in Hox genes, tissue-specific methylation patterns are targeted for histone modifications [23, 24]. This interplay between DNA methylation and histone modifications often exhibits a strong negative correlation, underscoring its significance in establishing distinct cell populations. The role of DNA methylation in adipogenesis can be observed at 3 stages of the process: (1) preadipocyte commitment, (2) early differentiation, and (3) late differentiation. However, how DNA methylation selectively regulates and changes its pattern during adipogenesis, thus leading to changes in gene expression, needs to be studied in detail [26]. Because the 3T3-L1 cell line exhibits a distinct and synchronized differentiation process from Pre-AD to fully grown, lipid-laden adipocytes that mimic preadipocyte differentiation in vivo, it is considered an appropriate model for studying adipogenesis. Environmental cues like diet and hormonal treatment initiate differentiation, causing or leading to significant chromatin remodeling and epigenomic changes, beginning 4 hours (4H) after induction and proceeding to TD [27-29]. Also, 2 distinct waves of transcription factors start off adipogenesis [27].

We used WGBS with 1 base resolution to demonstrate how genome-wide methylation patterns changed as 3T3 L1 cells differentiated by hormonal treatment. We focused on 4 essential time points: Pre-AD; day 0, representing the initial period when adipogenic factors are relatively inactive; 4H after differentiation induction, when the first wave transcription factors are highly active while the second wave transcription factors are expressed low; 2 days (2D), which marks the beginning of the elevated expression of the second wave of transcription factors and the initiation of terminal differentiation; and 15 days (15D), which displays the fully developed, mature, and lipid-rich adipocyte [30]. To our current understanding, this is the first attempt to analyze genome-wide methylation patterns in TD cells. Furthermore, we have looked for site-specific methylation at transcription factor hotspots where multiple transcription factors bind cooperatively and modify the structure of chromatin within hours after the induction of adipogenesis. Studying the epigenetic profile of Pre-AD and adipocytes can contribute to developing various treatments for obesity and metabolic disorders such as treating genetic disorders and other metabolic disorders through ex vivo gene therapy utilizing Pre-AD [31]. Unrevealing the mechanisms by which DNA methylation regulates differentiation will improve our understanding of the underlying biological pathways.

## Materials and Methods

### Chemicals and Reagents

DMEM and fetal bovine serum (FBS) were purchased from Gibco, lnc. (Grand Island, NY, USA). Isobutylmethylxanthine, dexamethasone, insulin, and 3-(4, 5-dimethylthiazol-2-yl)-2, 5-diphenyl tetrazolium bromide were obtained from Sigma (St. Louis, MO, USA). The EZ DNA Methylation Kit from Zymo Research was used for the bisulfite treatment of DNA (Zymo Research, Cat. No. D5001, USA).

### 3T3-L1 Preadipocytes Culture and Differentiation

3T3-L1 Pre-AD obtained from NCCS (Pune, India) were cultured in DMEM and supplemented with 10% FBS. The cells were maintained at 37 °C in a humidified atmosphere with 5% CO_2_. The cells were seeded at a density of 1 × 10^5^ cells/mL in a 6-well culture plate. After 2 days of reaching confluence, cell differentiation was induced by treating the cells with a differentiation medium containing 10% FBS DMEM supplemented with an MDI hormone cocktail (0.5 mM 3-isobutyl-1-methylxanthine isobutylmethylxanthine, 1 μM dexamethasone, and 1.0 μg/mL insulin). The medium was then replaced with 10% FBS DMEM containing 1.0 μg/mL insulin. Finally, the differentiation medium was replaced with 10% FBS DMEM. The 3T3-L1 Pre-AD were divided into 4 groups based on the postinduction time: undifferentiated Pre-AD, 4H and 2D postinduction, and 15D postinduction when cells are considered fully or terminally differentiated.

### Genomic DNA Isolation

The cells were harvested and washed twice with cold PBS. Subsequently, the cell pellets were snap-frozen in liquid nitrogen for storage at −80 °C or further processed. For DNA extraction, the frozen cell pellets were thawed at room temperature and resuspended in PBS following the instructions provided by the manufacturer (DNeasy Blood & Tissue Kits, Cat. No. 69504). Extracted DNA was further checked for quality on gel and subsequently bisulfite-treated and was used for WGBS sequencing and cloning of CpG-rich regions and DMRs.

### PCR Amplification, Hotspot Cloning, and Bisulfite Treatment of Samples for Sequencing

Hotspots were cloned to examine their DNA methylation states as the cells differentiate. Purified genomic DNA from undifferentiated and differentiated adipocytes was subjected to bisulfite modification using the EZ DNA Methylation kit (Zymo Research). Approximately 0.5 to 1 μg of genomic DNA was treated with bisulfite and eluted in 20 μL elution buffer following the manufacturer's protocol. After bisulfite treatment, 2 μL of the eluted DNA was amplified for 40 cycles using methylation-specific primers according to standard protocols. The PCR products were visualized by agarose gel electrophoresis and extracted from the gel using a gel extraction kit (Qiagen, Cat. No. 286040). The PCR products obtained from the gel were then cloned into the pcDNA plasmid as BamHI-XhoI fragments. Plasmid DNA was isolated from individual clones using the QIAprep Spin miniprep kit (Qiagen, Cat. No. 27106), and the cloned plasmids were subjected to sequencing using T7 forward primer and SP6 reverse primer designed for the vector backbone. The sequencing data provided information on cytosine methylation at each CpG site within the amplicon. The chromatograms obtained from sequencing were analyzed using Snapgene software, and the sequencing data were further analyzed for DNA methylation using BIQ Analyser software.

### Library Preparation for WGBS

High-quality genomic DNA was extracted using standard phenol/chloroform extraction, ethanol precipitation, or the DNeasy Blood and Tissue kit. The Pre-AD and adipocytes were lysed in lysis buffer at 37 °C for 1 hour and then digested with proteinase K at 10 μg/mL concentration for 3 hours at 50 °C. Following cell lysis, DNA was isolated using the phenol-chloroform extraction method. Eurofins Genomic India Pvt. Ltd. provided bisulfite conversion and sequencing services. To confirm the efficiency of bisulfite conversion, lambda DNA spike-in was added, and it was found that 99% of the DNA was successfully bisulfite converted. For library construction, 100 ng of genomic DNA was treated with the EZ DNA Methylation-Gold kit (Zymo Research) for bisulfite conversion. The resulting libraries, consisting of DNA fragments with lengths between 200 and 400 bps, were subjected to 150 bps pair-end sequencing on an Illumina platform. All sequencing analyses were performed based on the *Mus musculus* NCBI GRC38 genome assembly (mm10 version). The sequencing statistics can be found in Supplementary Fig. S1 [32]. The raw WGBS data (FASTQ and bedGraph files) is deposited in the NCBI SRA database (Bioproject code-PRJNA1034485).

### RNA Isolation and Quantitative Real-time RT-PCR

To identify differentially expressed genes, primers (Supplementary Table S1) [32] were designed using primer designing tools [33]. β-actin was considered an internal control to normalize gene expression signals. Three biological replicates and 2 technical replicates for each stage were run along with internal control in quantitative RT-PCR (qRT-PCR). Standard manufacturer protocol was used for the qRT-PCR reactions. Total RNA was extracted from 3T3-L1 Pre-AD, 4H postdifferentiation, 2D postdifferentiation, and 15D TD cells by TRIzol reagent (Ambion, USA). The purity and concentration of isolated RNAs were determined using the NanoDrop spectrophotometer (Thermo Fisher Scientific, USA). cDNA was synthesized by the iScript™ cDNA synthesis kit (Bio-Rad Laboratories, Inc). The mRNA expression of 45 adipogenic genes, ie, KLF5, KLF6, STAT5a, ZFP423, ZFP467, Tcf7l1, KLF2, Foxo1, Foxa2, Foxc2, Cd36, Lpl, Fasn, Plin1, Plin2, Plin3, Plin4, Plin5, DGAT1, ANGPTL4, PDGFRα, PDGFRβ, VEGFc, VEGFb, EGR2, Resistin, Lipoproteinlipase, FABP4, CREB1, TET1, TET2, TET3, ADIPOQ, GATA2, EBF1, HOXA6, HOXA5, VDR, KLF4, ATF7, JUNB, PBX1, Slc2a1/GLUT1, and KLF14, was evaluated using real-time PCR on CFX96 Real-Time system with SYBR Green Fast qRT-PCR mix from Bio-Rad. The reaction protocol involved priming at 25 °C for 5 minutes, reverse transcription at 46 °C for 20 minutes, and real-time inactivation at 95 °C for 1 minute. The gene expression levels were calculated using the normalized relative quantification protocol followed by the 2^−ΔΔCT^ method.

### WGBS Data Analysis

The quality of raw sequences was examined with FastQC (v0.11.9), and Trimmomatic (v0.39) [34] was used to remove the Illumina adaptor sequences and to filter out the low-quality reads and bases (Phred quality score < 15) using “SLIDINGWINDOW:4:15 LEADING:3 TRAILING:3 MINLEN:36 HEADCROP:10 ILLUMINACLIP: TruSeq3-PE.fa:2:30:10” parameters and the results are shown in Table 1. Following, clean reads were mapped to the mm10 (GRCm38) reference genome using Bismark (v0.23.1) [35]. The lambda genome (GenBank: J02459.1) was also mapped along with the reference genome to determine the bisulfite conversion efficiency (Table 1), (Supplementary Fig. S1) [32]. The obtained bisulfite conversion rate for CG context was above 99% for all libraries (all samples).

**Table 1.** The paired-end mapping with Bismark was performed with Bowtie 2 using the following parameters: –score_min L, 0, −0.6 -X 1000, and duplicated reads are removed using the deduplicate_bismark command (Supplementary Fig. S1C)

Samples	Raw reads	Clean read	Clean bases (G)	Clean ratio (%)	Mapped reads	Mapping rate (%)	Duplication rate (%)	Bisulphite conversion rate (%)
Pre-AD	55 438 326	53 411 178	14.5	87.2	39 346 076	73.7	19.9	99.3
4H	51 941 175	42 412 105	11.6	74.6	31 456 049	74.2	18.1	99.3
2D	55 463 283	53 311 291	14.4	86.7	45 153 417	84.7	18.5	99.4
15D	52 949 471	49 723 797	13.4	84.2	36 544 292	73.5	23.5	99.2

The genome-wide cytosine analysis was performed using the remaining reads; its results are given in Supplementary Fig. S1D. The methylation bias in the reads was determined with the -mbias option of Bismark Methylation Extractor; consequently, methylated CpGs were extracted by ignoring one nucleotide of 3′end of both reads along with -no-overlap -comprehensive -bedGraph -cytosine_report options.

Abbreviations: 2D, 2 days; 4H, 4 hours; 15D, 15 days; Pre-AD, preadipocytes.

The Pearson's correlation coefficient of Pre-AD and remaining samples was obtained using the MethylKit R package (v1.20.0) [36]. DMRs between control (Pre-AD) and 4H, 2D, and 15D were detected using the DSS package (2.42.0) [37]. In the pairwise comparison of control vs rest, DMLtest function with a smoothing span of 100 bps was applied to estimate mean methylation levels, and the callDMR function was used to detect DMRs having minimum 3 CpG sites, methylation difference >20%, minimum 50 bp length, and *P*-value < .05. Further, the DMRs, which are 100 bp apart, were merged.

For downstream analysis, the obtained DMRs were categorized into hypo- and hyper-methylated [Gene Ontology (GO) and Kyoto Encyclopedia of Genes and Genomes (KEGG) analysis]. The annotation was performed with ChIPseeker R package (v1.30.3) [38] and RefSeq mm10 annotation (http://hgdownload.cse.ucsc.edu/goldenpath/mm10/bigZips/genes/). The annotatePeak function of ChIPseeker was used to annotate hypo- and hyper-DMRs by defining the promoter as 3 kb upstream of the transcription start site (TSS). The obtained annotated genomic features, such as promoter, UTRs, exons, introns, and intergenic regions, were used for comparison and visualization. GO enrichment analysis of genes whose promoter overlapped with DMRs was performed by R package clusterProfiler (v4.2.2) with enrichGO function. The GO terms were determined based on the default Benjamini–Hochberg (BH) procedure and a cutoff score of adjusted *P*-value < .01 or q-value < 0.05, depending on the selected parameters. Further, enrichKEGG of clusterProfiler function is used for pathway analysis with the BH procedure (final parametric values were produced with fixed *P*-value cutoff = 1, p-AdjustMethod = “BH”, minGSSize = 1, maxGSSize = 500, q-value cutoff = 1).

### Genomic Region Analysis

The RefSeq genes annotation of mouse reference genome mm10 (GRCm38) was obtained from UCSC (https://hgdownload.soe.ucsc.edu/goldenPath/mm10/bigZips/genes/), and the mouse genome was divided into 9 regions. To avoid redundancy for protein-coding genes with multiple transcripts, only the longest was used for defining the locations of promoters, TSS (transcription start site), TES (transcription end site), exons, introns, and intergenic regions [39, 40]. Promoters are described at 0 to 3000 bases upstream of the TSS, 5′ UTR between the TSS and ATG start site, gene body between ATG and stop codon, all exons in the gene body, first exon of gene body, all introns in the gene body, first intron in the gene body, 3′UTR between the stop codon and poly-A site (or end of TSS), and intergenic regions as remaining regions between 2 genes [41]. Additionally, genomic locations of CpG islands and RepeatMasker were downloaded from the “UCSC table browser” (https://genome.ucsc.edu/cgi-bin/hgTables). The regions associated with CpG islands (CGI) were also explored by considering both shores (0-3000 bp in the upstream and downstream of CGI) and shelves (3000-4000 bp) in the upstream and downstream of CGIs.

DeepTools suite (3.5.1) [42] was used to generate and plot average methylation levels of different genome regions. The computeMatrix scale-regions were used to measure mean methylation levels across nonoverlapping windows with the following parameters: --binSize 10 --numberOfProcessors 40 --regionBodyLength 3000 -b 2000 -a 2000 for promoters and -b 0 -a 0 for other genomic elements or features. The plotProfile was used to compute the data matrix required for visualization. The bedGraph files obtained from the Bismark methylation extraction step were converted into bigwig format using UCSC bedGraphToBigWig for processing in DeepTools. Circos and Gene chromosome plots were made using LaTex with in-house scripts.

### Statistical Analysis

Data was analyzed using Excel and GraphPad Prism and presented as mean ± SEM. *P* < .05 was considered significant.

## Results

### DNA Methylation Profile During Pre-AD Differentiation.

**Figure 1.**
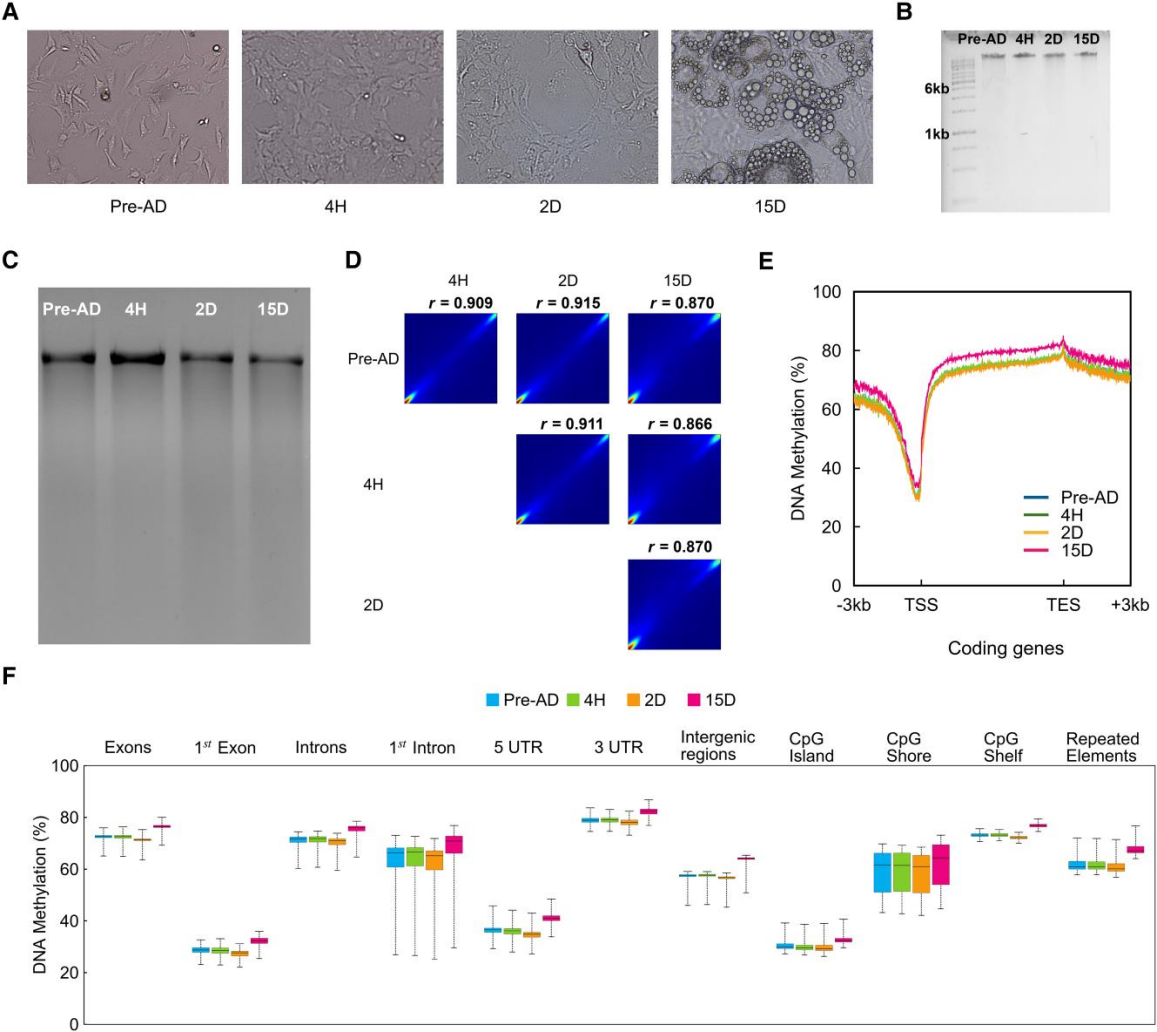
DNA methylation pattern during differentiation of preadipocytes to mature adipocytes. (A) Bright-field images depicting control 3T3-L1 preadipocytes and cells exposed to hormone cocktail to induce differentiation. Panels show preadipocytes, 4H, and 2D postinduction and 15D postinduction and TD cells. The presence of conspicuous lipid droplets characterizes TD cells. (B) Genomic DNA extracted from 3T3-L1 cells shows genome integrity during the differentiation process. (C) McrBc restriction digestion of genomic DNA extracted from preadipocytes and 4H, 2D, and 15D postinduction suggest genome-wide loss and gain of DNA methylation. (D) Pearson's correlation coefficient analysis of DNA methylation between preadipocytes and differentiating adipocytes and TD cells. (E) The DNA methylation levels with reference to the Transcription Start Site and Transcription End Site in coding transcripts. Traces for the control sample are superimposed by 4H and 2D sample traces and are not shown. (F) The levels of DNA methylation at various genomic annotations such as exon, intron, 5′UTR, 3′UTR, CpG islands, CpG shores, and CpG shelves. RefSeq mm10 annotations were used to obtain transcripts. Promoters are defined by considering 3 kb upstream regions. The bin size is 5, and the minimum base level depth of CpGs is 1. Abbreviations: 2D, 2 days; 4H, 4 hours; 15D, 15 days; Pre-AD, preadipocytes; TD, terminally differentiated.

We have used the homogenous NIH 3T3L1 cells in studying DNA methylation and cell differentiation since the cells are widely used as a preadipocyte proxy to study the complex phenomena of cell differentiation and underlying molecular mechanisms of adipogenesis in vitro. These fibroblast-like cells derived from the clonal expansion of the 3T3 Swiss albino cell line are already committed to the adipocyte lineage. Therefore, the NIH 3T3L1 Pre-AD cell line is commonly used to analyze the subcellular pathways involved in Pre-AD cell differentiation. Furthermore, due to its adipogenic pathway resembling human Pre-AD and its comparable sensitivity to different substances and nutrients, the cell line is utilized as an adipogenic model for primary human preadipocyte studies [43-45]. While in vitro systems may not fully replicate the complexity of in vivo conditions, they serve as valuable tools for hypothesis testing, drug discovery, and mechanistic studies. Data analysis revealed that DNA methylation exhibits changes throughout the adipocyte cell lineage, occurring during and after the differentiation process. To investigate the whole-genome DNA methylome profiles associated with lineage-specific adipogenesis, 3T3-L1 cells were cultured and induced to differentiate from Pre-AD to mature adipocytes in vitro. We performed WGBS on 3T3-L1 Pre-AD and differentiated cells by extracting and analyzing genomic DNA at 4H, 2D, and 15D postdifferentiation (Supplementary Fig. S2) [32]. Images of undifferentiated and differentiated adipocytes are shown (Fig. 1A).

Genomic DNA samples were treated with the McrBc enzyme to assess global DNA methylation levels in both undifferentiated and differentiated cells by targeting methyl CpG-rich regions for cleavage (Fig. 1B and 1C). At 4H, the genomic DNA band is intense compared to the faint band of undifferentiated and postdifferentiated 2D and 15D samples. This observation is interpreted as depicting the hypomethylation of the genome at 4H postdifferentiation. Examining the experiment's fidelity and the sample selection's rationality is crucial, and 1 key indicator is the correlation of methylation levels across samples. The Pearson correlations between samples vary from R^2^ = 0.86 to 0.91, suggesting a strong correlation and lack of any substantial changes in DNA methylation among different samples (Fig. 1D) except TD cells in which methylation is more pronounced.

WGBS analysis indicated that the overall global DNA methylation was similar between Pre-AD and adipocytes (Fig. 1E). Within the vicinity of the TSS, a valley depicting the loss in methylation was observed in all 4 samples (Fig. 1E). In contrast, higher DNA methylation was observed in gene body regions (Fig. 1F), a common feature observed in various cell types [18, 19, 46]. We compared in-house (NABI dataset) WGBS data analysis with the adipogenic reprogramming dataset [47] to validate our WGBS data further (Supplementary Material) [32] depicting comparative analysis and correlation analysis between AR and NABI datasets (Supplementary Table S3, Supplementary Figs. S6 and S7, Supplementary Table S4) [32]. The genome-wide distribution of DNA methylation exhibited similar patterns before and after the differentiation of adipocytes [48]. Nevertheless, when specifically considering methylated CpGs, a decline in trend was noted during adipocyte differentiation at the initial stages, suggesting DNA methylation is reduced in a restricted number of specific regions, indicating a regulatory role [49].

Furthermore, when whole genome regions were categorized as per different genomic annotations, Pre-AD and adipocytes exhibited variable DNA methylation patterns (Fig. 1F). Also, the methylation pattern in all the chromosomes was congruent with the methylation levels in the coding regions (Supplementary Fig. S3) [32]. The intergenic regions showed a substantial increase in the DNA methylation level at 15D (Fig. 1F). The process of terminal differentiation involves activating specific transcription factors and epigenetic modifications that regulate gene transcription, leading to the establishment of distinct cell fates [14]. To investigate the active demethylation process in 3T3-L1 Pre-AD, we compared the methylation levels between undifferentiated cells and differentiated cells. We classified a CpG site as demethylated if its methylation level decreased by more than 0.1 between the 2 compared stages, with statistical significance determined by Fisher's exact test (*P*-value < .05; false discovery rate < 10%). These results indicate active demethylation at many CpG sites during the transition from Pre-AD to mature adipocytes.

Our analyses revealed that some specific CpGs in Pre-AD are methylated after differentiation. Furthermore, a significant portion of highly methylated CpGs was found in introns, repeat regions, and gene bodies (Fig. 1E). In contrast, most unmethylated CpGs were located in promoters and CGIs (Fig. 1E and 1F), suggesting the importance of maintaining these regions in an unmethylated state for gene expression. We also demonstrated that non-CpG cytosine methylation is also dynamic during preadipocyte differentiation (Supplementary Fig. S1D) [32]. Figure 1F illustrates the average methylation levels of various functional genomic elements in Pre-AD and adipocytes. Such an analysis revealed significant demethylation in several functional elements, including CGIs and 5′ UTRs. A similar DNA methylation pattern was observed in the chromosome-wise plot (Supplementary Fig. S3) [32]. Interestingly, it was further observed that the methylation status of CGIs near the TSS remained stable. In contrast, CGIs within genic regions displayed greater dynamism during the early stages of differentiation [50-52]. Our study offers insights into the DNA methylation patterns associated with lineage-specific adipogenesis, highlighting the dynamic nature of DNA methylation and its potential role in regulating gene expression during adipocyte differentiation.

### Site-specific Methylation Pattern at Hotspots

**Figure 2.**
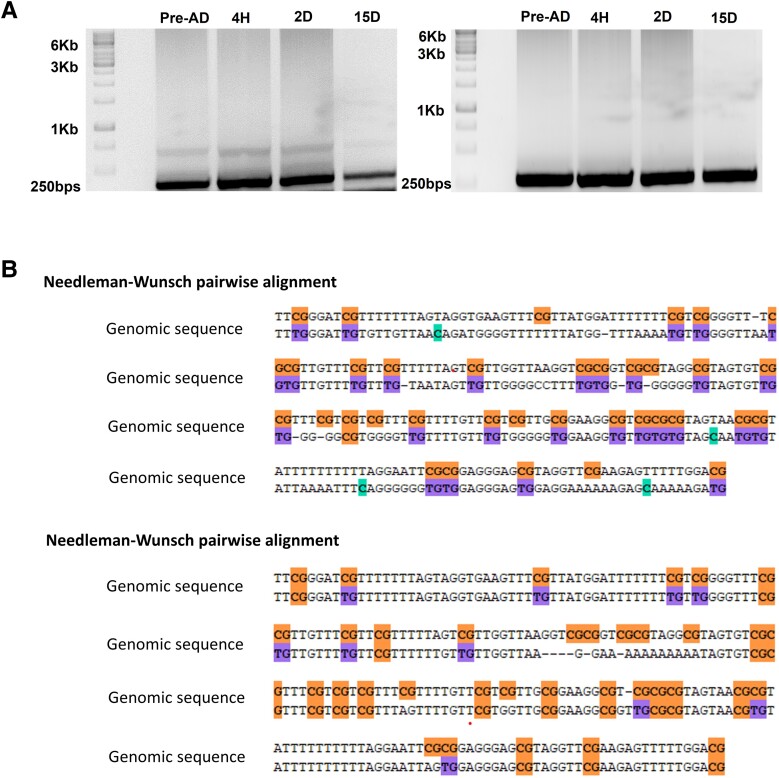
PCR amplification of hotspots at chromosomes 5 (chr5) and 8 (chr8). Methylation-independent primers were designed for the DNA methylation status of hotspots (250 bps) present at chr8: 19784539:19784789 and chr5: 13993644:139936704 using the Bisearch tool. (A) PCR amplified hotspots were bisulfite treated, then cloned in pcDNA plasmid and Sanger sequenced. (B) Sanger sequence analysis of Bisulphite treated cloned hotspot with BIQanalyzer depicting the change in methylation pattern at specific sites.

Genome-wide DNA methylation pattern demonstrated variability. To check DNA methylation at specific locations that acted as hotspots, ie, have sites for multiple transcription factors were evaluated for varying degrees of methylation at different time points used in this study. For the analysis of site-specific methylation, CpG-rich hotspots on chromosome 5 and chromosome 8 were PCR amplified (Fig. 2A), bisulfite-treated, cloned, and sequenced (Fig. 2B). It further demonstrated that as the cells differentiated, the methylation pattern changed in the hotspot regions containing binding sites for adipogenic transcription factors [53]. This confirmed that cytosine methylation at 4 indicative periods was dynamic at the genome and site-specific level, emphasizing the importance of cis-elements DNA methylation in gene regulation [54, 55].

### Characterization of DMRs

DMRs are contiguous genomic regions with variable DNA methylation levels that differ between phenotypes [56, 57]. DMRs can be found across the entire genome, but they are specifically recognized in and around gene promoter regions, within the gene bodies, and at intergenic regulatory regions [2, 58-64]. These genomic regions are considered potential functional regions involved in the transcriptional control of genes since they exhibit varying levels of methylation across various samples (tissues, cells, etc.) [65]. Finding DMRs across several tissues may reflect the epigenetic basis of gene regulation between tissues and cells [49]. Numerous DMRs have been identified during developmental reprogramming stages [66]. Here, DMRs between control (Pre-AD) and 4H, 2D, and 15D were identified using the DSS package (2.42.0).

**Figure 3.**
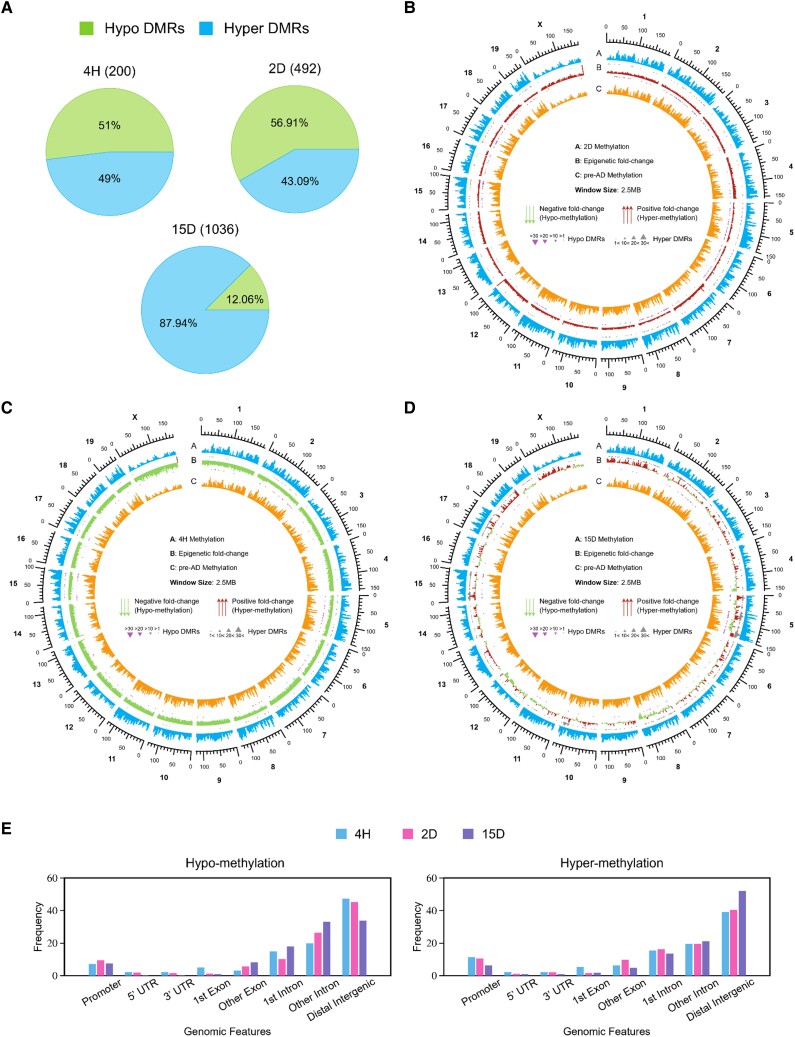
Analysis of DMRs present at different time points postdifferentiation compared to undifferentiated cells. (A) Pie chart depicting the difference between methylation level in hypo- and hyper-DMRs at the 3 different time points: 4H, 2D, and 15D compared to control. (B-D) Circos plot depicting fold change analysis of DMRs (B) control vs 2D (C) control vs 4H (D) control vs 15D. (E) Percentage of hypo-DMRs and hyper-DMRs at different time points postdifferentiation at various genomic annotations. Windows: 5MB Overlapping: 2.5M Base Level Depth: 1. Abbreviations: 2D, 2 days; 4H, 4 hours; 15D, 15 days; DMR, differentially methylated region.

By analyzing a subset of known and unknown DMRs in 3T3-L1 cells, we identified a small proportion that exhibited changes in DNA methylation during differentiation (Fig. 3). DMRs were categorized into hypo- and hyper-DMRs based on varying DNA methylation [66-68]. Comparing undifferentiated 3T3-L1 cells with 4H, 2D, and 15D fully differentiated cells, we found 200, 492, and 1036 novel DMRs, respectively. Furthermore, hypo-DMRs were dominant compared to hyper-DMRs at 4H and 2D postdifferentiation, ie, 51% and 56.9% (Fig. 3A). At 15D postdifferentiation, the hyper-DMRs were predominant (88%) compared to hypo-DMRs (Fig. 3A). We also compared the DNA methylation fold change in DMRs control vs 4H, 2D, and 15D (Fig. 3B–3D). The circos plot demonstrated that at 4H postdifferentiation, there was a pronounced hypomethylation (negative fold change), which suggests a significant reduction in methylation upon differentiation initiation. However, at 2D, there was substantial positive fold change depicting regain of DNA methylation. Furthermore, DMRs' status varies. The significant fold change in hypo-DMRs was observed at chromosome 15 at 4H (Fig. 3C), whereas significant changes were observed at chromosomes 2, 6, 5, 8, 11, 15, and 17 at 2D (Fig. 3D) on chromosomes 5, 10, and 15. However, after 15D, the methylation level increased, indicating that hyper-DMRs are not uniformly distributed across all chromosomes. To summarise, the DMR distribution, whether hypo or hyper, is not uniform and is biased for a few chromosomes (Supplementary Fig. S5) [32]. Also, genomic annotations show that a significant fraction of the hyper-DMRs were enriched in distal intergenic regions (Fig. 3E). The majority of DMRs were found in intronic and intergenic regions. In promoter regions, most DMRs were hypomethylated after 4H and hypermethylated after 15D.

The study implies that the methylation alterations observed during differentiation were unidirectional and transient and involved both hypermethylation and hypomethylation. Also, prominent hypomethylation was seen at the induction of differentiation. These findings imply a complicated and dynamic underlying process involved in adipogenesis that depends on CpG methylation. Also, DMRs have an active role in gene regulation.

### Correlation Between Hotspots and DMRs

**Figure 4.**
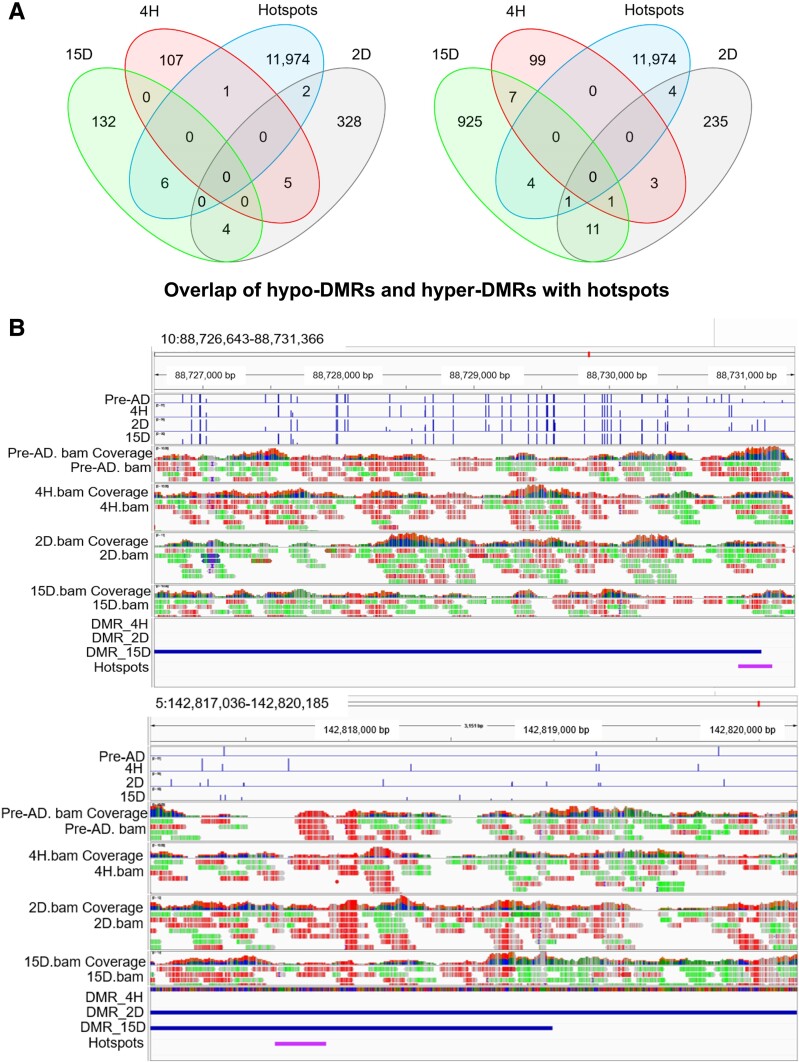
Hotspots are part of DMR. (A) Venn diagram showing overlap patterns of DMRs and hotspots at indicated time points of differentiation. (B) Representative genome browser view of overlap of Hotspots and DMRs using IGV, which depicts hotspots as a part of DMRs. Abbreviations: DMR, differentially methylated region; IGV, integrative genome viewer.

We further overlapped the locations of hotspots and DMRs to understand the role of DMRs in gene regulatory activities. DMRs-containing binding motifs of major transcription factors that are part of hypo-DMRs and hyper-DMRs were investigated. To characterize DMRs of undifferentiated and differentiated adipocytes in an unbiased manner, the regions were subdivided into various genomic annotations such as 5′-UTR, 3′-UTR, CpG island, CpG shore, exons, and introns (Fig. 3E). Few of the DMRs were part of hotspots, which suggests the regulatory role of DMRs during Pre-AD differentiation (Fig. 4). A total of 200 (98 hyper-DMR; 102 hypo-DMR), 492 (212 hyper-DMR; 280 hypo-DMR), and 1036 DMRs (911 hyper-DMRs; 125 hypo-DMRs) were found by WGBS at 4H, 2D, and 15D, respectively and are found to overlap with 11 974 hotspots (Fig. 4A and 4B).

### DNA Methylation Variability in Promoters

**Figure 5.**
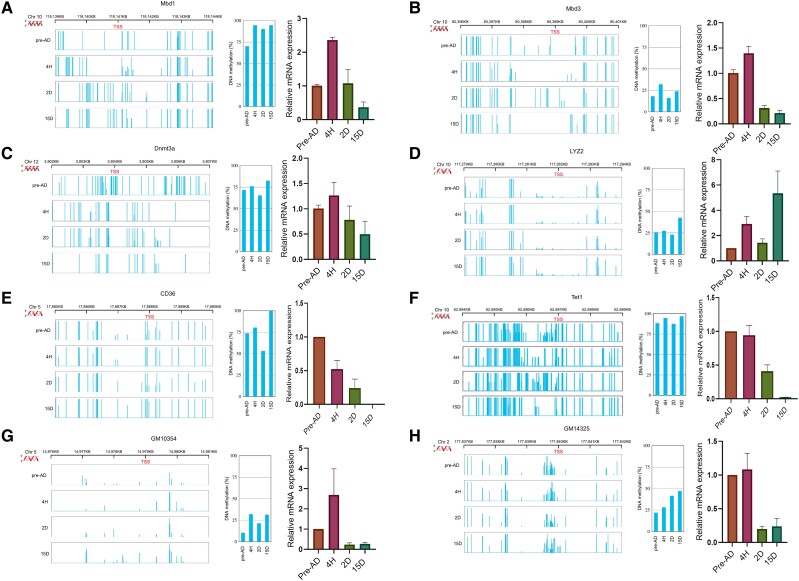
Illustrative presentation of a genome browser view displaying DNA methylation pattern in the promoters of genes (A) MBD1, (B) MBD3, (C) DNMT3A, (D) Lyz2, (E) CD36, (F) Tet1, (G) GM10354, (H) GM14325, and average DNA methylation level at the promoters of these genes along with genes expression profiles at indicated time points.

To investigate the probable mechanism for selective up- or downregulation of DNA methylation at loci unique to adipocytes, an integrative genome viewer (IGV) was used to evaluate the promoter DNA sequences of adipogenic genes showing complete coverage during 15D of terminal differentiation; there was an upturn in the average DNA methylation at the promoters of the genes *MBD1, MBD3, DNMT3A, LYZ2, CD36*, *TET1*, *GM10354,* and *GM14325* (Fig. 5). In previous studies and based on qRT-PCR expression analysis here, entrenched upregulation of DNA methylation often leads to gene repression. However, in the case of Lyz2, upregulation in DNA methylation led to increased gene expression, suggesting a direct relationship or positive correlation exists between DNA methylation and gene expression. Lyz2 is responsible for lysozyme expression in 3T3-L1 cells, which maintains the expression of genes related to adipogenesis and adipocyte differentiation [69]. Similarly, as reported earlier, we observed elevated gene expression and elevated methylation in the VDR and EBF1 promoter regions on 15D cells [47].

### Identification and Validation of Differentially Expressed Genes

**Figure 6.**
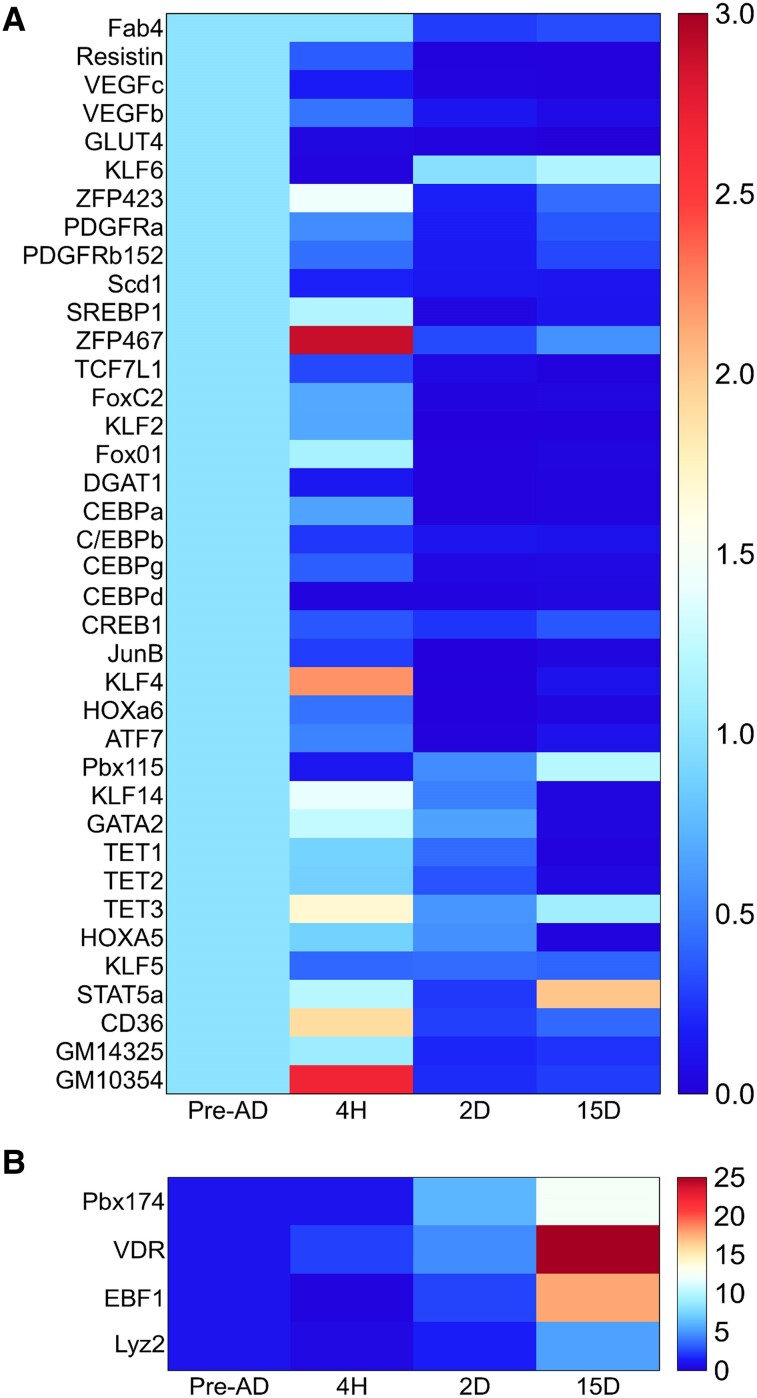
Using quantitative RT-PCR, heatmaps of gene expression values depicting clustering of genes among undifferentiated and differentiated cells based on mRNAs expression for a set of genes (*P*-value < .05). Samples are represented in rows, and genes are in columns. (A) Heatmap depicts the genes with up to 3-fold change in expression. (B) Genes with more than a 5-fold change in expression.

**Figure 7.**
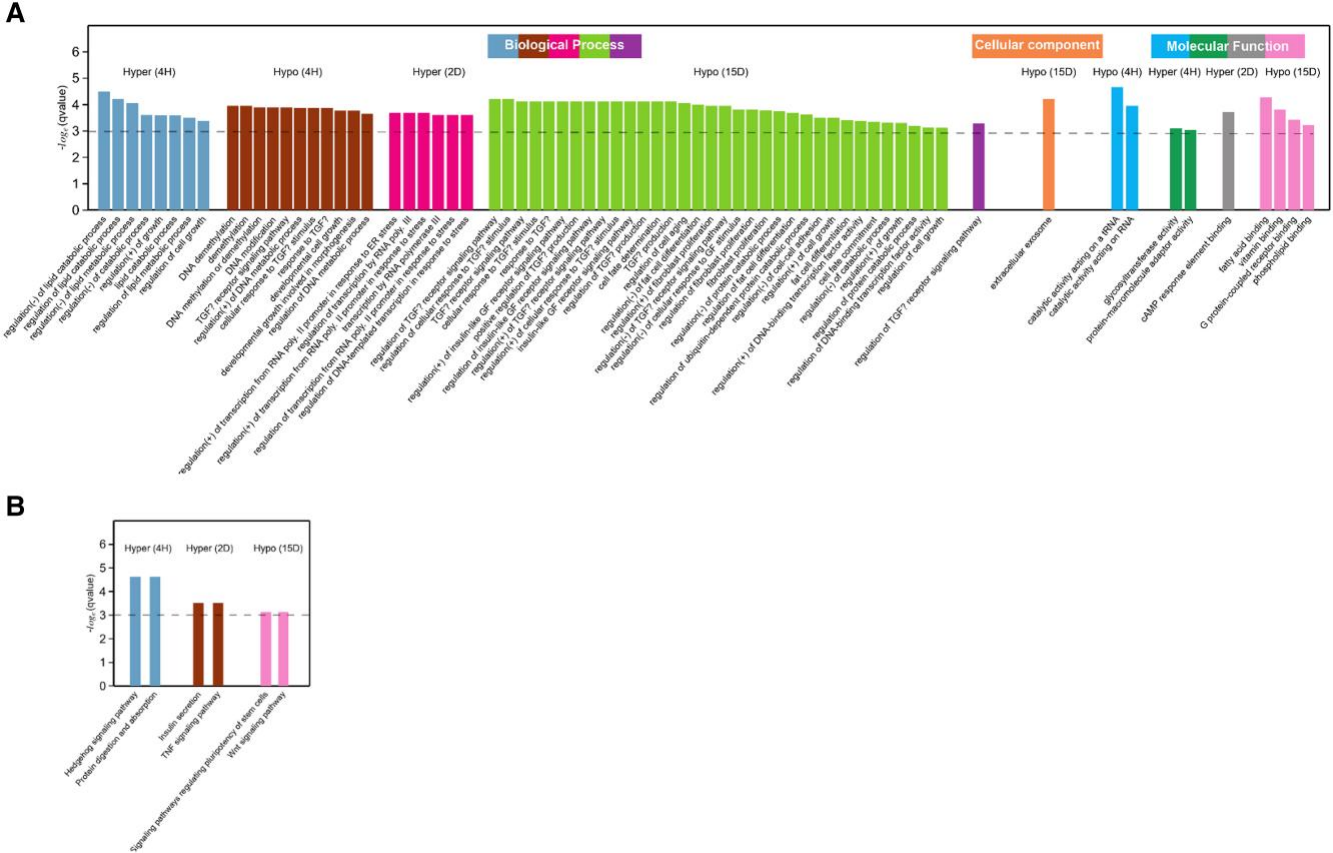
(A) Gene Ontology (B) Kyoto Encyclopedia of Genes and Genomes pathway recruitment analysis of differentially expressed genes in the differentially methylated regions: undifferentiated and differentiated cells were categorized into various functional groups: biological process, cellular component, and molecular function.

The comparative cycle threshold method was used to determine the fold change of the studied genes at each differentiation stage [70] (Fig. 6 and Supplementary Fig. S4) [32]. One-way ANOVA was used to determine statistical differences between maturation stages (*P-value < .05). The adipogenic genes *ZFP467, Tet3, KLF4, CD36,* and *GM10354* were expressed 2 to 5 fold at initial differentiation. However, *Stat5a, VDR, EBF1, Lyz2,* and *Pbx174* showed an exponential increase in expression at terminal differentiation (15D). qRT-PCR expression was performed for other adipogenic genes *DNMT3A, DNMT3B, DNMT1, MBD1, MBD2, MBD3, MBD4, MeCP21, MeCP2, UHRF1, UHRF2, CBX5, ZFP467, TCF7L1, FOXC2, KLF2, VDR, FOXO1, DGAT1, CEBPβ, KLF5, KLF6, STAT5a, EBF1, ZFP423, PDGFRα, PDGFRβ152, Pbx115, Fab4, Resistin, VEGFc, VEGFb, CD36, GLUT4, Scd1, SREBP1*that show inverse relation between methylation and gene expression as cells differentiate (Fig. 6, Supplementary Fig. S4) [32]. The result showed a 3-fold increase in *TET3* transcription increased during initial differentiation. However, *TET1* and *TET2* do not show any statistically significant change in expression [71]. We performed GO analyses using a list of genes with differentially methylated promoter regions [21, 23, 24]. Interestingly, the GO enrichment results aligned with our observations, suggesting that genes with hypomethylated promoters at 4H and 2D were associated with adipogenic processes (Supplementary Table S2) [32]. At the same time, genes with hypermethylated promoters at 15D were enriched with GO terms related to white fat cell differentiation, brown fat cell differentiation, regulation of lipid metabolic processes, IL-7 signaling, TGF-β signaling pathway, DNA replication, insulin-like factors, regulation of localization, and adipogenesis genes (Fig. 7A). Genes with hypomethylated promoters were identified in specific gene regions, indicating a link between DNA methylation reprogramming and Pre-AD differentiation. At 15D hyper-DMR, pathways associated with Lyz2, such as lysozyme activity and peptidoglycan muralytic activity, are upregulated. Upregulated expression of Lyz2 during 3T3L1 differentiation maintains the expression of genes associated with adipogensis and the differentiation of adipocytes [69]. This suggests that DNA methylation rapidly modifies gene loci following exposure to differentiation stimuli, eventually leading to gene expression variation.

### GO and KEGG Analysis

The interplay between cell cycle regulators and differentiation factors triggers a chain of events leading to the adipocyte phenotype [72]. Adipogenesis is a multistage process characterized by a specific gene expression pattern [20, 26, 47, 73, 74]. In the process of adipocyte differentiation from pluripotent stem cells, there are 2 distinct stages. The initial phase, termed determination, encompasses the commitment of pluripotent stem cells to Pre-AD. Pre-AD may exhibit morphological similarities to their precursor cells but undergo a loss of versatility to differentiate into alternative cell types. In the subsequent phase, referred to as terminal differentiation, Pre-AD progressively assume the feature of mature adipocytes and attain functional capabilities, including lipid transport and synthesis, insulin sensitivity, and the secretion of adipocyte-specific proteins [20, 25-27]. During the terminal differentiation stage, there is a notable surge in the newly formed synthesis of fatty acids. Transcription factors collaborate with genes associated with adipocytes, working in tandem to maintain the progression of precursor adipocytes into fully mature adipocytes [30, 75, 76].

To comprehend the roles of differentially expressed genes (DEGs), we conducted GO enrichment analysis to investigate their participation in biological processes, cellular components, and molecular functions (Fig. 7A). DEGs were significantly enriched in metabolism-related pathways, including white cell differentiation, brown cell differentiation, TGF-β signaling, lipid metabolic pathways, interleukin signaling, DNA replication, fat cell differentiation, insulin-like growth factors, and steroid metabolic processes. We conducted a GO enrichment analysis to examine the biological significance of the DEGs in adipocytes. Among the biological processes, many DEGs were involved in cellular and metabolic processes. Most DEGs were associated with cell components and organelles in the cellular component category. Regarding molecular function, many DEGs were linked to catalytic activity and transcription regulator activity. This analysis helps to explain the functional roles and relationships of the DEGs in DMRs in adipocyte biology.

### KEGG Pathway Analysis of DEGs

We exploited the KEGG pathway database to explore the signaling pathways associated with the DEGs in adipogenesis. This analysis revealed specific components that play a role in adipogenesis. Among the top 20 significant pathways of DEGs, the most prominent enrichments were observed in metabolic pathways, lipid biosynthesis, and the steroid metabolism pathway [47]. On the other hand, the TNF signaling pathway, hedgehog signaling pathway, insulin secretion, protein digestion and absorption, signaling pathways regulating pluripotency of stem cells, and Wnt signaling pathway were predominantly enriched (Fig. 7B). This analysis provides valuable insights into the molecular mechanisms and biological processes involved in adipogenesis and helps to identify key pathways that may regulate the differentiation and function of adipocytes.

Identifying and verifying key genes regulating adipogenesis is crucial for understanding the molecular mechanisms underlying adipocyte differentiation. We compared DEGs in DMRs between the control and 3 experimental groups to identify potential candidate essential genes that regulate adipogenesis in 3T3-L1 cells [22, 77, 78]. This study reveals that groups' differences in gene expression are linked to the differentiation of fat cells, lipid accumulation, and insulin production in treated 3T3-L1 cells. DNA methylation may also impact gene expression associated with these pathways.

### Dynamic DNA Methylation at Promoter of Lyz2

This study found that the Lyz2 gene's promoter region was hypermethylated in terminally differentiated adipocytes (Supplementary Fig. S8) [32]. The CGs present at promoter regions depicted a substantial change in DNA methylation. Beyond that, CpG methylation was dynamic. Hypermethylation at promoter region at TD has been reported in some cases previously and its causal role in disorders [79, 80].

This is the first study to demarcate dynamic DNA hypermethylation at the promoter of Lyz2 gene during differentiation of Pre-AD indicating the causal role of DNA methylation at Lyz2 promoter that is deemed to be of great relevance.

### Non-CpG DNA Methylation

The present study focused on variations in the methylome across the entire genome at the single CG dinucleotide resolution of Pre-AD and adipocytes. However, some non-CpG changes have been observed [81, 82]. Variable non-CpG methylation, ie, CHH and CHG, are observed. In the case of Pre-AD, the CHH and CHG were around 0.86 and 0.85, respectively, which subsequently decreased at 4H to 0.83 and 0.82, respectively. Cytosine methylation in non-CG sites further increased to 0.94 and 0.95 at 15D, demonstrating the dynamic variations in non-CpG methylation as the differentiation of Pre-AD progressed (Supplementary Fig. S1) [32]. This aspect of Pre-AD differentiation may be further studied to establish the role of non-CpG methylation in Pre-AD differentiation.

## Discussion

Emerging data indicates that cellular identity is also determined by a distinct DNA methylation pattern [48, 66, 74, 83-85]. The function of DNA methylation in 3T3L1 cell differentiation has been investigated here, as the precise regulatory effects and underlying mechanisms on Pre-AD’s transformation into adipocytes are still unknown. To provide insight into these DNA methylation-mediated regulatory processes, we employed Pre-AD as a model system to see dynamic DNA methylation patterns as cells proceed to differentiate. Employing WGBS methodology, the study revealed the DNA methylation during different time periods. Previous studies have focused on earlier time periods ranging from a few hours to 6 days. To our knowledge, this is the first attempt to investigate high-resolution genome-wide DNA methylation at time intervals of 4H, 2D, and 15D, later corresponding to the Pre-AD’s terminal differentiation.

Here, we have presented a comprehensive bisulfite sequencing workflow and analysis for 3T3L1 cells (Supplementary Fig. S2) [32]; at the early stages, ie, during initial differentiation of Pre-AD, DNA methylation was found to be significantly reduced at CpG sites as well as non-CpG sites. However, as the differentiation progresses, the DNA methylation pattern is regained in the later stages, ie, terminal differentiation. We also highlight varying global DNA methylation patterns and “hotspots” site-specific variability. Transcription factor hotspots show cooperative binding of multiple transcription factors and restructure the chromatin structure within hours after induction of adipogenesis [28, 53, 85]. Analysis of CpG-rich hotspots on chr5 and chr8 and their DNA methylation status at 4 different time points suggests the importance of DNA methylation in gene regulatory functions. DMR analysis reduces the likelihood of negative random associations compared to single CpG site-based data [4, 86]. We found a subset of known and unknown DMRs in 3T3L1, which exhibited changes during the differentiation of Pre-AD. Novel DMRs with adipogenic genes were found. We discovered around 200, 492, and 1036 novel DMRs at 4H, 2D, and 15D. Furthermore, hypo-DMRs were pre-dominant at 4H and 2D post-differentiation. At 15 days, the hyper-DMRs were enriched. Also, the idiogram of DMRs (Supplementary Fig. S5) [32] confirmed that the distribution of DMRs was not uniform but was rather restricted to specific chromosomes. To further understand the role of DMRs in gene regulatory functions, hotspots overlapped with DMRs. In some cases, there was a partial overlap between hotspots and DMRs, whereas in others, complete overlap confirmed the correlation of gene regulatory functions with DMRs.

It is suggested that upregulation of DNA methylation at promoters leads to the repression of genes. On the contrary, DNA methylation at promoters can increase gene expression (this study and [25]). We found that DNA methylation at promoters of *MBD1, MBD3, DNMT3A, LYZ2, CD36, TET1, GM10354,* and *GM14325* was upregulated at 15D; however, the gene expression was decreased in all the cases except LYZ2, GM10354, and GM41325 where the DNA methylation and gene expression was positively correlated. At terminal differentiation, expression of VDR, EBF1, GM14325, LYZ2, GM10354, and C/EBPγ was enhanced.

**Figure 8.**
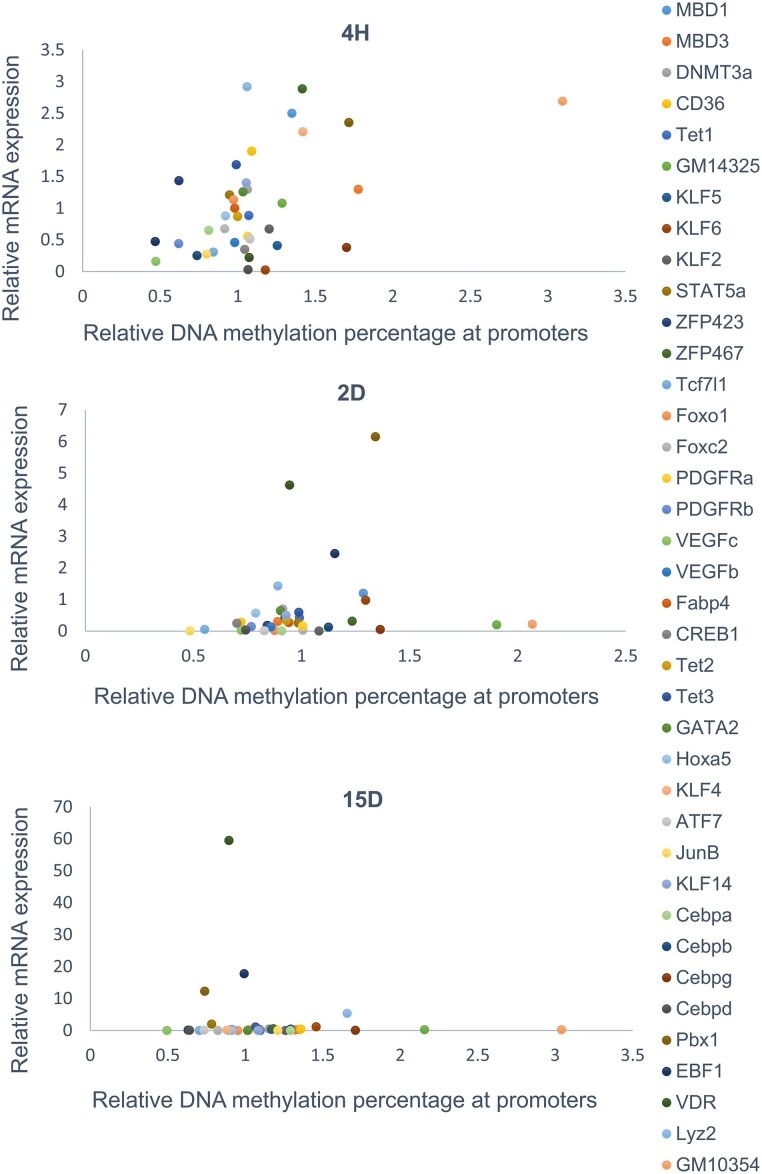
The connection between DNA methylation and gene expression was examined by plotting matched methylation and gene expression data obtained through bisulfite sequencing of NIH 3T3L1 and quantitative RT-PCR. The x-axis illustrates the extent of methylation at CG sites within the promoter, while the y-axis represents relative mRNA expression for the selected genes.

Most adipogenic genes indicate decreased gene expression with increased promoter methylation, with few exceptions where an inverse relation is observed at the initiation of terminal differentiation and terminal differentiation (Fig. 8). Therefore, it is suggested that the relationship between promoter methylation and gene activity is complex and context-dependent. Further, key transcription factors and DEGs are significantly upregulated or downregulated, and the methylation status of key transcription factor promoters was altered. These findings provide valuable insights into regulating various adipocyte-specific genes during the process of adipogenesis.

Furthermore, to ascertain the potential connection between alterations in DNA methylation and cellular functions, we conducted GO and KEGG analyses on genes exhibiting DMRs to examine enriched pathways. Genes featuring hypo-DMRs were linked with fat cell differentiation, DNA methylation and demethylation, cell fate determination, fibroblast proliferation, extracellular exosome, fatty acid binding, GPCR binding, and phospholipid binding. However, the hyper-DMRs were associated with lipid catabolic process and cAMP response element binding.

Our study has contributed to a deeper understanding of adipogenesis mechanisms while identifying potential epigenetic targets for regulating this process. The comparative analysis of hyper- and hypo-DMRs at 2D and 15D (Supplementary Table S5) [32] signifies that at terminal differentiation some pathways are part of hyper-DMRs such as positive regulation of mitotic nuclear division, positive regulation of glucose metabolic process, glycogen biosynthetic process, and glucan biosynthetic process. In contrast, hypo-DMRs consist of pathways such as regulation of fat cell differentiation, regulation of protein localization to the nucleus, and regulation of osteoblast differentiation.

Our study has demonstrated a dynamic DNA methylation pattern during Pre-AD differentiation. We have shown loss of DNA methylation at initial differentiation and regain of methylation pattern at the terminal stage of differentiation. This work has investigated the epigenetic mark DNA methylation associated with the differentiation of Pre-AD and its association with relevant genes associated with adipogenesis. The promoter of lyz2 showed increased methylation and gene expression at terminal differentiation.

Mouse NIH3T3-L1 cell line is established to follow adipogenesis in vitro. Although studies involving this cell line have helped decipher the gene regulation and many physiological pathways involved in preadipocyte differentiation, 3T3-L1 cells have limitations. Notably, the hormonal cocktail used here has a high concentration of dexamethasone (1 μM), which does not mimic the in vivo glucocorticoid concentrations. Furthermore, although NIH 3T3L-L1 mimics human Pre-AD, still, it may not represent the situation in vivo where the Pre-AD are regulated by exogenous and endogenous factors such as glucocorticoids, sex steroids, insulin, sex-related differences, and various pathophysiological conditions. Future studies with Pre-AD derived from various fat depots exposed to true physiological conditions will shed more light on the methylation-dependent differentiation of these cells.

In summary, cytosine methylation, as an epigenetic mechanism, regulates gene expression during adipogenesis. Understanding the dynamic changes in DNA methylation and its impact on adipocyte differentiation and function is crucial in unraveling the molecular mechanisms underlying obesity and related metabolic disorders.

## Conclusion

DNA methylation plays a significant role in the lineage-specific development of adipocytes. Hypermethylation occurs during terminal differentiation, suggesting DNA methylation's role in maintaining mature adipocytes. Few gene promoters, when hypermethylated, show high gene activity. Therefore, DNA methylation-dependent gene expression is context-dependent. Further understanding of the regulatory role of non-CpG methylation and targets for dedifferentiation can offer a more thorough insight into the epigenetic control involved in the differentiation of adipocytes.

## Data Availability

The raw Whole Genome Bisulphite Sequencing Data was deposited in the National Centre for Biotechnology Information Sequence Read Archive under Bioproject code PRJNA1034485 https://dataview.ncbi.nlm.nih.gov/object/PRJNA1034485?reviewer=6ftoba5lhh750m05u25ovdlqmf. The *Mus musculus* mm10 data was used as the reference for alignment with raw data.

## Code Availability

The method presented in the paper and the code of this study is shared via zenodo [32].
